# Editorial: Neurodegeneration, cell signaling and neuroreparative strategies

**DOI:** 10.3389/fphar.2022.1059923

**Published:** 2022-10-19

**Authors:** Ana Rita Vaz, Ana Sofia Falcão, Valle Palomo

**Affiliations:** ^1^ Neuroinflammation, Signaling and Neuroregeneration Group, Research Institute for Medicines (iMed.ULisboa), Faculty of Pharmacy, Universidade de Lisboa, Lisbon, Portugal; ^2^ Department of Pharmaceutical Sciences and Medicines, Faculty of Pharmacy, Universidade de Lisboa, Lisbon, Portugal; ^3^ iNOVA4Health, NOVA Medical School, Faculdade de Ciências Médicas, NMS, FCM, Universidade Nova de Lisboa, Lisboa, Portugal; ^4^ Instituto Madrileño de Estudios Avanzados en Nanociencia (IMDEA-Nanociencia), Madrid, Spain; ^5^ Centro de Investigación Biomédica en Red de Enfermedades Neurodegenerativas (CIBERNED), Instituto de Salud Carlos III, Madrid, Spain

**Keywords:** neurodegenerative diseases, CNS-intracellular and extracellular communication, neuroinflammation and immunoregulation, therapeutic applications, paracrine signaling

Neurodegenerative diseases are usually characterized by the accumulation of misfolded proteins and malfunction of proteasomal, autophagosomal, lysosomal or mitochondrial systems, that lead to a progressive loss of vulnerable populations of neurons in specific regions of the central nervous system (CNS). Glial cells and neuroinflammation are key contributors for the neurodegenerative process but their precise role remains unclear. A better understanding of the cell-cell communication will contribute to unveil the pathophysiological mechanisms that trigger neurodegeneration and will help to find specific targets for modulation. Non-cellular players, including the overall secretome and the extracellular vesicles (EVs), including the small EVs (sEVs), also designated as exosomes, may be also important contributors for the maintenance and spread of pathological processes that depend on cell-to-cell communication, and may also constitute targets for modulation. Indeed, they are important in the intercellular communication due to their capacity to transfer multiple different messengers, such as proteins, lipids, nuclei acids or microRNAs.

This Research Topic gives an overview of the neuronal-glial pathophysiological mechanisms involved in several existing models of neurodegenerative diseases. In addition, new potential therapeutic strategies targeting either intercellular and intracellular communication are proposed for the several existing models of neurodegenerative diseases, through 10 articles by 51 authors, which contains 3 reviews and 1 mini-review, as well as 5 original research papers and 1 brief research report, (Total views: 21,535; as of 30 Sep 2022).

One of the reviews focuses on the recent progress in methods for manufacturing, isolation and engineering sEVs that may be used as a therapeutic strategy to overcome neurodegeneration in CNS pathologies, namely in models of Alzheimer’s and Parkinson’s Disease, Amyotrophic Lateral Sclerosis and Brain Tumours (Loch-Neckel et al.). Another review discusses the cell non-autonomous proteostasis function and decline along the aging process, by focusing on the systemic activation and mechanisms of the heat shock and unfolded protein responses of the endoplasmic reticulum and mitochondria, as well as transcellular chaperone signaling and transcellular transfer of proteotoxic material (Ferreira et al.). The third review focuses on the molecular mechanisms underlying ischemic stroke and presents the new systemic glutamate scavenging methods that may be used in combination or as an alternative to the current available drugs and therapeutic approaches for stroke patients (Kaplan-Arabaci et al.). There is also a mini-review that examines the pathophysiological mechanisms of spinal cord injury, emphasizing the important contribution of the inflammatory processes, and how its inhibition by Safflower Yellow, an HMGB1-TLR4-NF-κB signaling pathway inhibitor, have a reparative effect in injured spinal cord tissue (Wang et al.).

This Research Topic also contains original studies covering critical aspects of neurodegeneration and putative therapeutic strategies. Among them, two studies focused on the contribution of myelinating cells in models involving neurodegeneration. In the first one, the benefit of basic fibroblast growth factor (bFGF) to promote the facial nerve repair after injury was explored (Hu et al.). The Authors developed a thermosensitive *in situ* forming poloxamer hydrogel that was used as a vehicle to deliver bFGF for treating facial nerve injury (FNI) in rats. Data indicate that the bFGF-hydrogel promote facial nerve regeneration by promoting autophagy and inhibiting apoptosis. They also demonstrated that such repair is mediated by the PAK1 signaling pathway activation in Schwann cells, which may provide a promising strategy to be used for FNI recovery. The other study addressed the protective effect of GABA receptors agonists, namely baclofen and muscimol, against AMPA-induced excitotoxicity in cultured rat oligodendrocytes (Bayon-Cordero et al.). They found that GABA receptors activation initiates alternative molecular mechanisms that attenuate AMPA-mediated apoptotic excitotoxicity in oligodendrocytes, and propose GABA receptor agonists as potential oligodendroglial protectants in CNS disorders.

In another study, the neuroprotective effects of entinostat, a class I histone deacetylases (HDAC) inhibitor were studied in a model of spinal cord injury (SCI) (Dai et al.). For that, a mouse model of SCI and an *in vitro* model of oxygen-glucose deprivation (OGD) were used. The authors concluded that entinostat suppressed HDAC activation and also improved the motor function, histopathological damage, local inflammatory response and NLRP3 inflammasome activation in the spinal cord following SCI. Entionostat also had a neuroprotective role in the OGD-induced neuronal damage *via* the NLRP3 inflammasome, highlighting the interaction between the HDAC and NLRP3 inflammasome in the pathologic process of SCI.

Finally, three studies were focused in neurodegenerative disorders, namely using Alzheimer’s Disease (AD) and Amyotrophic Lateral Sclerosis (ALS) models. Regarding AD, the first study aimed to determine whether the regulation of Rho protein kinase 2 (Rock2) by the activator of the E3 ubiquitin ligase anaphase-promoting complex/cyclosome (APC/C), Cdh1, is an important mechanistic event in AD (Lapresa et al.). For that, Authors used an oligomerized form of the amyloid-beta (Aβ) peptide (Aβ25-35) that was incubated in neuronal primary mice cultures and also injected intracerebroventricularly in the mice, to induce an *in vitro* and an *in vivo* AD model, respectively. Results identified a novel Cdh1-Rock2 pathway that is involved in Aβ-mediated neurotoxicity, which may open novel therapeutic opportunities for AD. The other study on AD used a co-culture system of two human cell lines, microRNA (miR)-124 modulated SWE SHSY-5Y-neurons and CHME3-microglia stimulated with interferon-gamma (IFNγ), to determine how miR-124 modulation in SWE cells influences microglia polarized subtypes in the context of inflammation (Garcia et al.). The authors found that SWE miR-124 inhibitor favored IFNγ-induced inflammatory signature in microglia, while the SWE miR-124 mimic reduced their activation. Microglia proteomics also identified 113 responsive proteins to SWE miR-124 levels, including a subgroup of proteins involved in immune function/inflammation. This study was innovative in suggesting that neuronal miR-124 reshapes microglia plasticity, highlighting the contribution of neuronal survival to the inflammatory mechanisms that occur in AD-associated pathophysiology. In the study regarding the ALS model, the authors compared the potential therapeutic effects of three Sigma-1 receptor (Sig-1R) ligands, namely the agonists PRE-084 and SA4503 and the antagonist BD1063, in the SOD1G93A mouse model of ALS (Gaja-Capdevila et al.). They found that PRE-084 and BD1063 treatment could preserve neuromuscular function of the hindlimbs and increased the number of surviving motor neurons in the ALS mice, while SA4503 only slightly improved motor function, concluding that Sig-1R ligands are promising tools to consider for ALS treatment.

Overall, this Research Topic discussed a few proofs of concept that non-neuronal cells and their communication actively contribute to the degenerative process in several models of neurodegeneration and how the modulation of such communication mechanisms could uncover novel therapeutic strategies. Some of these strategies are here highlighted, providing valuable information for the development of innovative strategies designed to overcome the neurodegenerative process.

